# Predictive Value of Histopathological Variables and Peripheral Immune-Inflammatory Markers in De Novo Metastatic Breast Cancer: Development and Clinical Utility of the Novel KiTNASP Score

**DOI:** 10.3390/biomedicines14091960

**Published:** 2026-08-31

**Authors:** Serkan Yilmaz, Mesut Yur, Erhan Aygen, Yavuz Selim İlhan, Ahmet Akbaş, Şafak Özer Balin, Ali Rıza Avul

**Affiliations:** 1 Department of Surgical Oncology, Fırat University, Elazig 23240, Turkey; s_yilmaz@firat.edu.tr (S.Y.); eaygen@firat.edu.tr (E.A.); yilhan@firat.edu.tr (Y.S.İ.);; 2 Department of Surgical Oncology, Karadeniz Technical University, Trabzon 61080, Turkey; ahmetakbas@ktu.edu.tr; 3 Department of Infectious Diseases & Clinical Microbiology, Fırat University, Elazig 23240, Turkey; safak@firat.edu.tr

**Keywords:** breast cancer, neutrophil, lymphocyte, alkaline phosphatase, metastasis

## Abstract

**Background and Aim**: Approximately 10% of breast cancer patients present with distant organ metastases at initial diagnosis. This study aimed to evaluate the efficacy of immune-inflammatory markers and histopathological variables in identifying de novo metastatic breast cancer. **Methods**: Patients with breast cancer referred to a tertiary surgical oncology clinic between January 2020 and December 2022 were retrospectively screened. A total of 412 patients met the strict inclusion criteria. Laboratory parameters and radiological findings obtained during the initial diagnostic workup, prior to the initiation of any therapeutic intervention, were comprehensively evaluated. **Results**: Among the screened cohort, 56 patients presented with synchronous distant organ metastases at diagnosis. Significant differences (*p* < 0.05) were observed between the metastatic and non-metastatic groups in serum hemoglobin, albumin, and alkaline phosphatase (ALP) levels, lymphocyte and neutrophil counts, hemoglobin-albumin-lymphocyte-platelet score, prognostic nutritional index (PNI), systemic immune-inflammation index (SII), monocyte-to-lymphocyte ratio, platelet-to-lymphocyte ratio, neutrophil-to-lymphocyte ratio, and pan-immune-inflammation value. Multivariate logistic regression analysis identified Ki-67, T stage, N stage, ALP, SII, and PNI as independent predictors of metastasis (*p* < 0.05). In the receiver operating characteristic (ROC) analysis, the prognostic score formulated from this predictive model demonstrated an area under the curve (AUC) of 0.821 (95% CI: 0.781–0.857, *p* < 0.001, and Z-score = 9.34). At an optimal cut-off value of >0.233, the score yielded a sensitivity of 64.3% and a specificity of 92.1%. Furthermore, Decision Curve Analysis (DCA) confirmed a positive net clinical benefit across the decision-making threshold. **Conclusions**: The developed prognostic score may be a promising clinical tool for differentiating de novo metastatic breast cancer from non-metastatic disease at initial staging. This non-invasive approach may help clinicians risk-stratify patients, reduce diagnostic delays, and optimize early intervention strategies. Nonetheless, larger prospective multicenter studies are warranted for robust validation.

## 1. Introduction

Breast cancer remains the most frequently diagnosed malignancy and a leading cause of cancer-related mortality among women worldwide [1]. Approximately 10% of patients present with synchronous distant organ metastases at the time of initial diagnosis, which automatically classifies the disease as Stage IV [2]. Although conventional imaging modalities, such as computed tomography (CT) and positron emission tomography-computed tomography (PET-CT), are routinely employed to detect distant dissemination, these procedures are inherently resource-intensive, costly, and time-consuming [3]. Consequently, there is a clinical need for cost-effective, rapid, minimally invasive blood-based biomarkers to identify high-risk patients during the initial diagnostic workup.

To date, established clinicopathological factors—including primary tumor size (T stage), axillary lymph node involvement (N stage), histological type, lymphovascular invasion, human epidermal growth factor receptor 2 (HER2) status, and hormone receptor expression—have been extensively utilized as prognostic indices [4]. However, their predictive efficacy in precisely identifying systemic metastasis at the time of initial presentation remains poorly defined.

Concurrently, the tumor microenvironment and host systemic inflammatory responses have emerged as pivotal drivers of oncogenesis and metastasis [5]. Peripheral blood-based inflammatory indices, such as the neutrophil-to-lymphocyte ratio (NLR), systemic immune-inflammation index (SII), prognostic nutritional index (PNI), and pan-immune-inflammation value (PIV), have demonstrated significant prognostic value for overall survival (OS) and disease-free survival across multiple solid tumors, including breast cancer [6,7,8,9,10,11,12,13,14]. Nevertheless, whether a synergistic combination of these blood-based inflammatory parameters and standard histopathological variables can accurately predict synchronous de novo metastasis remains underexplored.

Additionally, serum alkaline phosphatase (ALP), an enzyme intrinsically linked to bone remodeling and hepatic function, has shown potential clinical relevance in metastatic malignancies. Elevated ALP levels may indicate liver disease or certain bone disorders. Although breast cancer bone metastases are typically osteolytic, the associated localized osteoblastic response can cause fluctuations in serum ALP levels. It is thought to modulate the tumor microenvironment, cytokine expression, cell proliferation, and migration [15,16]. Because altered systemic inflammation and nutritional depletion often coexist with advanced tumor burdens, a multiparametric approach that integrates these variables may yield higher diagnostic accuracy. Therefore, this study aimed to evaluate the combined efficacy of histopathological criteria, peripheral immune-inflammatory markers, and biochemical variables (ALP and albumin) in predicting de novo distant metastasis in breast cancer patients at initial presentation. Furthermore, we sought to develop and internally validate a novel clinical scoring model to optimize patient risk stratification and guide screening protocols.

## 2. Methods

### 2.1. Ethical Approval

This retrospective study was initiated after receiving approval from the ethics committee and administration. Approval from the Fırat University Non-invasive Ethics Committee (approval number: 2023/10/9) was obtained. The study was conducted in accordance with the Declaration of Helsinki. Written approval was obtained from Firat University Hospital, where the study was conducted, for the use of the data. The institution requires all patients to provide written consent before treatment, including for data use.

### 2.2. Patient Selection

**Figure 1:**
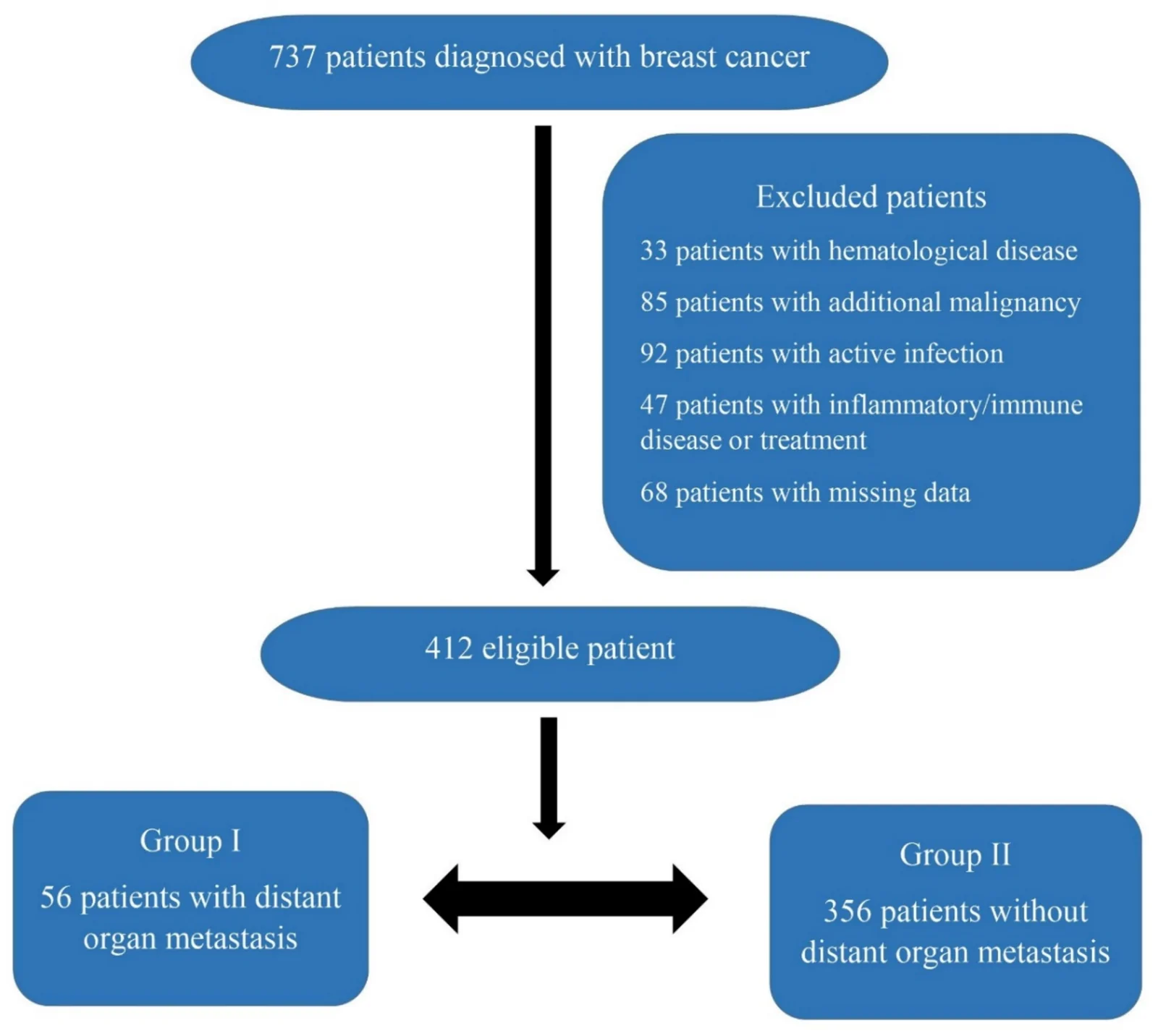
Flowchart of the patient selection process.

Patients referred to the surgical oncology clinic at Fırat University for follow-up or check-up for breast cancer between January 2020 and December 2022 were included in this study. All patients were newly diagnosed and treatment-naïve at the time of inclusion. We excluded patients who were male, had hematological diseases, additional malignancies, active (acute or chronic) infections, missing data before treatment, inflammatory diseases, or immunosuppressive treatment/diseases. The patients were divided into two groups: those with distant organ metastasis (Group I; M1) and those without (Group II; M0) (Figure 1).

Blood samples were collected after pathological diagnosis, prior to screening for distant metastasis. Hematological parameters included SII (neutrophil × platelet/lymphocyte), HALP (hemoglobin (g/L) × albumin (g/L) × lymphocyte/platelet, NLR (neutrophil/lymphocyte), MLR (monocyte/lymphocyte), PIV (neutrophil × platelet × monocyte/lymphocyte), PLR (platelet/lymphocyte), and PNI ((10 × albumin [gr/dL]) + (0.005 × absolute lymphocyte count [per mm^3^])).

### 2.3. Pathological Assessments

All pathological assessments were conducted centrally by the institution’s pathologists. The T-stage was regarded as the tumor diameter (T1: ≤ 2 cm, T2: > 2 cm– ≤ 5 cm, T3: > 5 cm, and T4 invasion of the skin and/or chest wall or inflammatory cancer).

Axillary lymph node status was evaluated in all patients using clinicoradiological or Tru-Cut biopsy findings. It was categorized as either metastatic or non-metastatic. The N-stage was regarded as non-metastatic (N0) or metastatic if at least one lymph node (N1–3) was involved.

#### 2.3.1. Estrogen Positivity

Cytoplasmic staining or <1% nuclear staining of cells was considered negative and ≥1% nuclear staining was considered positive in immunohistochemical staining [17].

#### 2.3.2. HER2 Status

Tumors with immunohistochemical staining scores of 3+ (uniform, intense membrane staining of 30% of invasive tumor cells) were considered HER2-positive. Cases with IHC staining scores of 2+ were considered positive if they turned out to be positive in subsequent HER2/neu gene amplification (fluorescence in situ hybridization). HER2 1+ status and the absence of staining were considered negative. Breast cancer is divided into five molecular luminal types [18].

### 2.4. Data Collection

Patient data that met the appropriate criteria were obtained from the hospital data system. All pathology data of the patients (hormone receptor status, Ki-67 expression, T-stage, lymph node status, grade, lymphovascular invasion), laboratory data (hemoglobin, lymphocytes, neutrophils, platelets, monocytes, albumin, glucose, alkaline phosphatase, and Ca^+^), and imaging for metastasis (abdominal and thoracic computed tomography or PET-CT images and any additional images if necessary) were obtained. Patients with missing data were excluded from the study.

### 2.5. Power Analysis

The power analysis was conducted as a post-hoc test (achieved power) using G-Power 3.1.9.7 statistical software (developed by Franz Faul, Universität Kiel, Kiel, Germany) because the predictive usefulness of the new scoring model on the probability of distant organ metastasis in breast cancer has not been previously studied in the literature. The effect size (w) was 1.67, and the power of the study (1-beta) was 1 for predicting distant organ metastasis in the metastatic breast cancer group, taking into account the estimated cut-off value, at an alpha error probability of 0.05 (Supplementary Materials).

### 2.6. Statistical Analyses

All data were analyzed using the SPSS 22 statistic software package. The normality of the data distribution was assessed using the Kolmogorov–Smirnov and Shapiro–Wilk tests. Parametric data are expressed as mean ± standard deviation (SD), and nonparametric data as median (minimum–maximum). The independent sample *t*-test was used to compare parametric data, whereas the Mann–Whitney U test was used to compare nonparametric data. The Chi-square or Fisher’s exact test was used to analyze categorical data. Logistic regression analysis (binary regression analysis) was used to predict the metastatic disease. All immune inflammation markers and histopathological data were determined by univariate analysis, and the variables with *p* values < 0.05 were entered into the multivariate analysis. The Forward LR method was used to determine the optimal predictive formula. The binary logistic regression formula for the prediction model was “Y = [(A × β^a^) + (B × β^b^) + (C × β^c^) + constant].” The optimal cutoff values for the predictive factors were determined using a receiver operating characteristic (ROC) curve. The area under the receiver operating characteristic curve (AUC ROC) was classified as follows: no discrimination for 0.5, acceptable for 0.7–0.8, excellent for 0.8–0.9, and outstanding for >0.9 [19]. The difference between the ROC curves was evaluated using the DeLong method. The Youden index was used to select the most suitable cutoff value. Decision curve analysis was performed using R (version 4.6.1) via RStudio (version 2026.08.1). The statistical significance level for all tests was set at *p* < 0.05.

## 3. Results

This study included 412 patients. The median age was 54 (23–88) years. Distant organ metastases were detected in 56 patients (Group I) at initial diagnosis. Of these, 43 had bone metastasis, 22 had lung metastasis, 15 had liver metastasis, 5 had brain metastasis, and 4 had metastases at other distant sites. While seven patients had a single metastatic lesion, the other 49 had multiple lesions. Table 1 shows the other demographic, pathological, and laboratory data.

Significant differences were observed between the groups in age, N-stage, T-stage, tumor diameter, Ki-67 level, histological type, hemoglobin, albumin, ALP, lymphocytes, neutrophils, NLR, SII, MLR, PLR, PNI, PIV, and HALP score (*p* < 0.05). No significant differences were observed between the groups in the other pathological and laboratory variables (Table 1).

**Figure 2:**
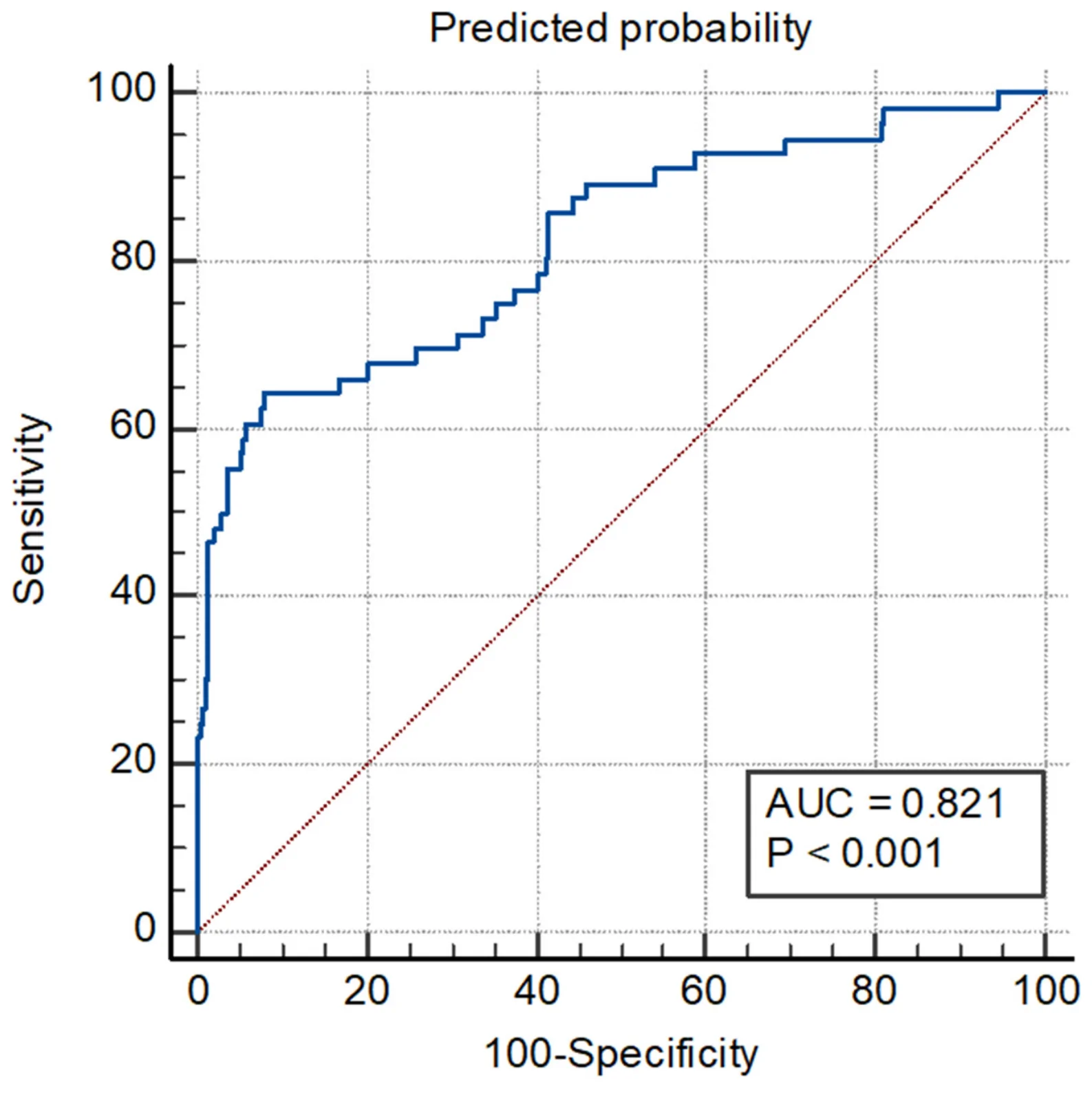
ROC curve for KiTNASP score. There was a statistically significant difference in the KiTNASP score compared to SII, NLR, HALP, PNI, MLR, PLR, and PIV (*p* < 0.05, AUC values: 0.641, 0.681, 0.630, 0.659, 0.631, 0.582, and 0.635, respectively).

**Figure 3:**
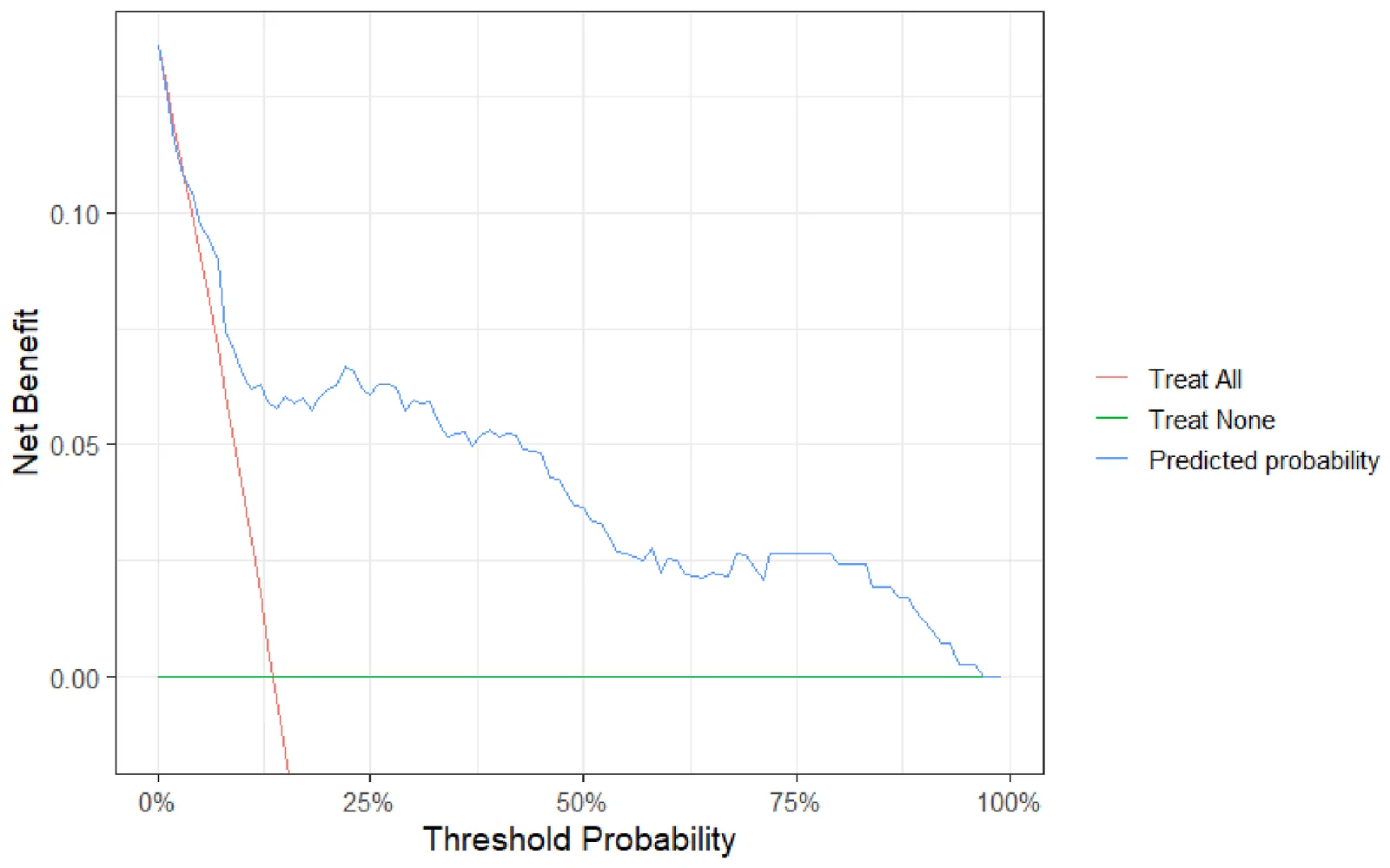
Decision Curve Analysis (DCA) evaluating the clinical utility of the developed prognostic score for predicting de novo metastatic breast cancer. The net benefit (*y*-axis) is plotted against a spectrum of risk threshold probabilities (*x*-axis). The blue curve represents the comprehensive predictive model and shows superior clinical net benefit over both the “Screen All” (red line) and “No Screening” (green line) strategies across nearly the entire decision-making threshold window (5% to 95%). This demonstrates the model’s practical value in optimizing patient risk stratification and reducing unnecessary diagnostic staging or over-treatment.

All immune inflammation markers and histopathological data were included in the univariate analysis. In the univariate analysis, hemoglobin, ALP, albumin, lymphocyte, neutrophil, HALP score, SII, NLR, PLR, MLR, PIV, and PNI values, Ki-67, T-stage, N-stage, and tumor diameter showed a significant difference (*p* < 0.05) (Table 2). Values that showed a significant difference in univariate analysis were included in multivariate analysis, and among these parameters, ALP (odds ratio (OR): 1.017, 95% CI; 1.009–1.026, *p* < 0.001), SII (OR: 1.001, 95% CI; 1.000–1.002, *p* = 0.012), PNI (OR: 0.856, 95% CI; 0.782–0.938, *p* = 0.001), Ki-67 (OR: 1.020, 95% CI; 01.005–1.035, *p* = 0.010), T-stage (OR: 3.786, 95% CI; 1.573–9.113, *p* = 0.003), and N-stage (OR: 4.687, 95% CI; 1.728–12.713, *p* = 0.002) were found to be risk factors. The VIF scores for ALP, SII, PNI, Ki-67, T stage, and N stage were 1.019, 1.028, 1.031, 1.018, 1.047, and 1.047, respectively. Probability values were calculated using the logistic regression formula based on Ki-67, T stage, N stage, ALP, SII, and PNI (KiTNASP score) [KiTNASP score = 1.762 + (Ki-67 × 0.019) + (T-stage × 1.27) + (N-stage × 1.627) + (PNI × −0.158) + (SII × 0.001) + (ALP × 0.018)]. The results obtained were evaluated using ROC analysis, and the AUC was found to be 0.821 (95% CI: 0.781–0.857, *p* < 0.001, and z-score: 9.34) (Figure 2). There was a statistically significant difference in the KiTNASP score compared to SII, NLR, HALP, PNI, MLR, PLR, and PIV (*p* < 0.05, AUC values: 0.641, 0.681, 0.630, 0.659, 0.631, 0.582, and 0.635, respectively). When the KiTNASP score was dichotomized using the cut-off value (>0.233), its sensitivity in detecting metastatic disease was 64.3% (95% CI: 50.4–76.6), and its specificity was 92.1% (95% CI: 88.8–94.7) (Table 3). The baseline prevalence of metastatic disease in our breast cancer cohort was 13.6% (n = 412). Decision curve analysis (DCA) demonstrated that the default ‘Treat All’ strategy provided a net benefit of 0.136 at a threshold probability of 0%, which expectedly decreased to 0.051 as the threshold increased to 9%. Notably, our proposed model outperformed both the ‘Treat All’ and ‘Treat None’ strategies across a wide and clinically relevant threshold probability range of [5%] to [95%], yielding the highest net benefit within this window (Figure 3, Table 4).

## 4. Discussion

This study evaluated the predictive value of histopathological variables and inflammatory markers for distant organ metastasis in breast cancer at the time of initial diagnosis. Our findings indicate that Ki-67, T-stage, N-stage, ALP, SII, and PNI serve as independent predictors of de novo breast cancer metastasis. Furthermore, the newly developed KiTNASP score demonstrated acceptable accuracy, as reflected by the ROC curve.

While traditional performance metrics like the AUC quantify diagnostic accuracy, they do not account for the clinical consequences of false-positive and false-negative decisions. In the context of metastatic breast cancer, where non-screening can precipitate rapid disease progression or inappropriate treatment, and screening all patients exposes them to unnecessary time loss, evaluating clinical utility is paramount. Using Decision Curve Analysis (DCA), we demonstrated the tangible clinical value of our model. In our cohort, with a 13.6% prevalence of metastatic events, a blanket “Screen All” approach quickly loses clinical justification as the risk threshold rises, yielding a net benefit of only 0.051 at a 9% threshold. Our model addresses this challenge by providing a significant net benefit precisely within the critical decision-making window of 5% to 95%. Implementing this model in clinical practice could optimize patient risk stratification, reduce screening of all patients, and ensure that patients at high risk for metastatic disease are identified early and referred to appropriate systemic therapies.

The most common sites of distant metastasis in breast cancer are the bones, lungs, liver, and brain. During the initial staging workup, screening modalities varied by patient: some underwent thoracic and abdominal CT, while others underwent PET-CT [20]. MRI was performed for cases with suspected central nervous system involvement [3]. Each imaging modality has inherent diagnostic limitations. For instance, PET-CT cannot accurately visualize brain metastases because the brain has high baseline metabolic activity. Furthermore, pulmonary nodules smaller than 10 mm may not show detectable metabolic activity, underscoring the rationale for the widespread use of hybrid PET-CT [21]. Consequently, certain lesions may be missed on standard CT scans or underdetected on PET-CT. Notably, the National Comprehensive Cancer Network (NCCN) guidelines do not mandate a single imaging modality for initial staging [3].

Neutrophils and lymphocytes, the primary cellular immune components, contribute to cancer metastasis through distinct biological mechanisms. An elevated neutrophil count, reflecting heightened systemic inflammation, has been documented across various malignancies, including breast cancer [22]. To stimulate neutrophilia, tumors secrete potent signaling factors, such as granulocyte colony-stimulating factor (G-CSF) and granulocyte-macrophage colony-stimulating factor (GM-CSF), which drive the expansion of granulocyte-monocyte and neutrophil progenitors [23]. Proliferating neutrophils promote metastasis by secreting immunosuppressive mediators and angiogenic factors—such as reactive oxygen species (ROS), matrix metalloproteinase 9 (MMP-9), and vascular endothelial growth factor (VEGF)—thereby suppressing CD8+ T lymphocytes [23]. This cascade ultimately facilitates tumor cell dissemination through localized immunosuppression. Our findings suggest that the elevated neutrophil counts in the metastatic cohort are driven by these interconnected pathways.

Lymphocytes are another critical immune population, and their prognostic relevance has been extensively studied in breast cancer. A decline in total lymphocyte count, particularly in the CD4+ subset, is strongly correlated with disease progression [23,24]. This lymphopenia may be driven by elevated plasma CCL22 levels, activation-induced cell death, and impaired thymic function in patients with advanced disease [25]. Interestingly, this CD4+ lymphopenia appears to be independent of age, despite the natural involution of the thymus in older individuals. In the present study, the lower lymphocyte counts observed in the metastatic group likely reflect these suppressive mechanisms.

The SII has emerged as a robust inflammatory marker linked to poor prognosis and reduced OS across various cancers. Studies have shown that, as in breast cancer, a high SII value is associated with short OS and poor prognosis [11,26,27,28]. One study found it to be an independent risk factor for prostate cancer metastasis at the initial diagnosis [29]. Consistent with the existing literature, our study confirms that a high SII value is independently associated with metastatic disease at diagnosis.

Alkaline phosphatase has also demonstrated strong predictive potential for breast cancer metastasis [30]. Although marked elevations in ALP are typically expected in patients with bone-dominant metastatic disease due to osteoblastic activity, elevated ALP levels have also been observed in patients without bone involvement [31]. Although breast cancer bone metastases are typically osteolytic, the associated localized osteoblastic activity can still elevate serum ALP. In this study, ALP levels were significantly higher in the metastatic cohort, confirming ALP as an independent predictor of de novo metastasis.

The PNI, calculated from serum albumin and peripheral lymphocyte counts, reflects the intersection of a patient’s nutritional status and immune competence. Lower PNI levels have been linked to lymph node involvement [32]. Moreover, it has been reported to have prognostic value for both survival and metastasis [33,34]. This study found that PNI is an independent risk factor for metastasis in patients with de novo breast cancer, and those with low PNI levels are more likely to have detectable metastatic disease.

The heightened metabolic demands of malignant tumors accelerate energy consumption, often culminating in cancer cachexia [35]. Breast cancer patients with low albumin levels were found to have a higher risk of metastatic disease [30]. Consistent with reports linking hypoalbuminemia to an increased risk of metastasis, serum albumin levels in our study were significantly lower in the metastatic group. This decline likely reflects the metabolic strain imposed by a higher tumor burden and multiple active metastatic foci.

The Ki-67 proliferation index is a well-established surrogate marker of tumor aggressiveness and recurrence risk. Previous studies have consistently linked elevated Ki-67 expression to a higher propensity for distant dissemination [36,37]. In our cohort, Ki-67 levels were significantly higher in the metastatic group, reinforcing the premise that high cellular proliferation drives aggressive tumor phenotypes and early distant metastasis.

In breast cancer, clinical staging advances as tumor diameter and nodal burden increase. Although Stage IV disease is defined by distant organ involvement regardless of the T or N stage, distant metastases are more common in patients with large primary tumors and extensive nodal involvement, as larger tumors have a higher propensity for lymphatic dissemination [36,38]. In our study, the metastatic cohort presented with significantly larger tumor diameters.

Axillary lymph node status remains a cornerstone of breast cancer evaluation. While primarily used to define locally advanced disease, it strongly indicates systemic spread, with distant metastases more common in node-positive cases [37,38]. In our study, lymph node metastasis was markedly enriched in the distant metastasis group. Both T and N stages are integral components of the TNM staging system, which effectively integrates anatomical extent with biological aggressiveness to predict clinical outcomes.

This study has several limitations that warrant consideration. First, its retrospective, single-center design and relatively small sample size introduce potential selection bias and lack the validation of a prospective cohort or external datasets. Second, formal survival analysis was not performed. The limited number of patients presenting with de novo metastatic disease (13.6%) reflects the natural epidemiology reported in the literature, where roughly 10% of patients present with synchronous metastasis at initial diagnosis. These factors may have caused unintentional biases. While standard metastatic screening was indicated for all patients, staging modalities were not perfectly uniform; some patients underwent PET-CT, whereas others were evaluated with conventional CT imaging, potentially introducing diagnostic heterogeneity. Furthermore, peripheral neutrophil and lymphocyte counts are dynamic parameters sensitive to confounding physiological and pathological states, such as an unknown infectious condition or subclinical infections. Finally, potential confounding factors, including metabolic disorders and detailed patient comorbidities, were not adjusting for or examined in the current analysis.

In conclusion, this study shows that Ki-67 expression, T-stage, N-stage, serum ALP levels, SII, and PNI are independent predictors of distant organ metastasis in patients with breast cancer. Furthermore, our clinical data demonstrate that the novel KiTNASP score reliably identifies synchronous distant metastasis during the initial diagnostic workup. By refining risk stratification, the KiTNASP score could enhance personalized clinical decision-making, enabling prompt therapeutic interventions for high-risk individuals. Although peripheral blood parameters cannot currently replace conventional staging imaging, integrating this score holds promise for reducing both financial burdens and diagnostic delays. Ultimately, multi-center prospective studies with larger cohorts remain essential to robustly validate the clinical utility of our findings.

## Figures and Tables

**Table 1:** Demographic, laboratory and pathological data of patients and groups.

Variable		Total n = 412 (100%)	Group I n = 56	Group II n = 356	*p* Value
Age (years)		54 (23–88)	48 (26–85)	55 (23–88)	0.049
Tumor side	Right	208 (50.5%)	32 (57.1%)	176 (49.4%)	0.122
	Left	198 (48.1%)	22 (39.3%)	176 (49.4%)	
	Bilateral	6 (1.5%)	2 (3.6%)	4 (1.1%)	
Menopausal status	Premenopausal	166 (40.3%)	27 (48.2%)	139 (39%)	0.241
	Postmenopausal	246 (59.7%)	29 (51.8%)	217 (61%)	
Histological type	IDC	348 (84.4%)	54 (96.4%)	294 (82.6%)	0.021
	ILC	39 (9.5%)	1 (1.8%)	38 (10.7%)	
	Mixed type	25 (6.1%)	1 (1.8%)	24 (6.7%)	
Estrogen receptor	Negative	87 (21.1%)	10 (17.9%)	77 (21.6%)	0.520
	Positive	325 (78.9%)	46 (82.1%)	279 (78.4%)	
Progesterone receptor	Negative	126 (30.6%)	19 (33.9%)	107 (30.1%)	0.559
	Positive	286 (69.4%)	37 (66.1%)	149 (69.9%)	
HER2	Negative	268 (65%)	35 (62.5%)	233 (65.4%)	0.667
	Positive	144 (35%)	21 (37.5%)	123 (34.6%)	
Ki-67 (%)		30 (1–95)	35 (2–95)	30 (1–95)	0.009
Luminal type	Luminal A like	57 (13.8%)	5 (8.9%)	52 (14.6%)	0.860
	Luminal B Her2 (−)	169 (41%)	24 (42.9%)	145 (40.7%)	
	Luminal B Her2 (+)	115 (27.9%)	17 (30.4%)	98 (27.5%)	
	HER2-like	29 (7%)	4 (7.1%)	25 (7%)	
	Triple-negative	42 (10.2%)	6 (10.7%)	36 (10.1%)	
T-stage	I and II	370 (89.8%)	40 (71.4%)	330 (92.7%)	<0.001
	III and IV	42 (10.2%)	28 (28.6%)	26 (7.3%)	
N-stage	N 0	149 (36.2%)	5 (8.9%)	144 (40.4%)	<0.001
	N1–3	263 (63.8%)	51 (91.1%)	212 (59.6%)	
M stage	M0	356 (86.4%)			
	M1	56 (13.6%)			
Tumor diameter (mm)		25 (1–125)	30 (6–109)	25 (1–125)	0.002
HALP score		39.53 (8.62–107.37)	32.27 (8.62–77.53)	40.29 (10.13–107.38)	0.002
NLR		2.23 (0.51–13.5)	2.89 (1.14–7.39)	2.15 (0.51–13.5)	<0.001
SII		627.39 (81.05–3388.62)	801.8 (139.16–2611.19)	599.72 (81.05–3388.62)	0.001
MLR		0.22 (0.04–1.25)	0.25 (0.10–0.74)	0.21 (0.04–1.25)	0.002
PLR		147.2 (50.2–563.4)	163.8 (50.2–443.4)	145.6 (56.5–563.4)	0.048
PIV		270.3 (29.2–2240.6)	349.6 (54.3–1592.7)	255.8 (29.2–2240.6)	0.001
PNI		44 (29–56.5)	42 (29–49)	44 (34–56.5)	<0.001
Hemoglobin (g/dL)		13.2 (7.1–16.8)	12.7 (7.1–15.8)	13.3 (8.1–16.8)	0.007
Albumin (g/dL)		4.4 (2.9–5.7)	4.2 (2.9–4.9)	4.4 (3.4–5.7)	<0.001
Lymphocytes (10 × 10^3^/μL)		1.92 (0.4–5.32)	1.71 (0.75–3.54)	1.96 (0.4–5.32)	0.011
Platelets (10 × 10^3^/μL)		278 (105–672)	276 (105–672)	278 (110–613)	0.909
Neutrophils (10 × 10^3^/μL)		4.26 (1.3–10.5)	4.9 (2.1–10.5)	4.1 (1.2–10.4)	<0.001
Monocytes		0.42 (0.08–1.5)	0.44 (0.24–0.95)	0.42 (0.08–1.5)	0.268
Glucose (mg/dL)		100 (61–334)	101 (71–312)	100 (61–334)	0.265
ALP (U/L)		76 (25–337)	95 (42–337)	75 (25–249)	0.001
Ca (mg/dL)		9.4 (6.5–11.1)	9.3 (7.1–11)	9.5 (6.5–11.1)	0.061
Bone metastasis	Nonmetastatic	369 (89.6%)			
	Metastatic	43 (10.4%)			
Lung metastasis	Nonmetastatic	390 (94.7%)			
	Metastatic	22 (5.3%)			
Liver metastasis	Nonmetastatic	397 (96.4%)			
	Metastatic	15 (3.6%)			
Brain metastasis	Nonmetastatic	407 (98.8%)			
	Metastatic	5 (1.2%)			
Other metastases	Nonmetastatic	408 (99%)			
	Metastatic	4 (1%)			
Metastasis count	None	356 (86.4%)			
	Single	7 (1.7%)			
	Multiple	49 (11.9%)			

Data are presented as median (range) or count (percentage) as appropriate.

**Table 2:** Univariate and multivariate logistic regression analyses of predictors for de novo metastasis.

Variable	Univariate Analyses		Multivariate Analyses	
	OR (95% CI)	*p* Value	OR (95% CI)	*p* Value
Hemoglobin (g/dL)	0.764 (0.639–0.913)	0.003		
Albumin (g/dL)	0.161 (0.074–0.352)	<0.001		
ALP	1.019 (1.012–1.027)	<0.001	1.017 (1.009–1.026)	<0.001
Lymphocytes (10 × 10^3^/μL)	0.543 (0.334–0.884)	0.014		
Neutrophils (10 × 10^3^/μL)	1.295 (1.095–1.532)	0.003		
Platelets (10 × 10^3^/μL)	1.001 (0.998–1.005)	0.465		
Monocytes	2.359 (0.470–11.834)	0.297		
HALP score	0.048 (0.007–0.325)	0.002		
NLR	1.317 (1.110–1.563)	0.002		
SII	1.001 (1.001–1.002)	<0.001	1.001 (1.000–1.002)	0.012
MLR	7.875 (1.386–44.730)	0.020		
PLR	1.004 (1.001–1.008)	0.011		
PIV	1.001 (1.001–1.002)	0.001		
PNI	0.833 (0.771–0.901)	<0.001	0.856 (0.782–0.938)	0.001
Ki-67 (%)	1.015 (1.002–1.027)	0.020	1.020 (1.005–1.035)	0.010
T stage	5.077 (2.511–10.264)	<0.001	3.786 (1.573–9.113)	0.003
N stage	6.928 (2.699–17.782)	<0.001	4.687 (1.728–12.713)	0.002
Tumor diameter (mm)	1.022 (1.008–1.036)	0.001		

**Table 3:** Cross-tabulation of KiTNASP score with distant metastasis at the cutoff value (sensitivity: 64.3%, specificity: 92.1%, PPV: 56.3%, NPV: 92.1%, and accuracy: 88.4%).

		M Stage		Total
		M0	M1	
KiTNASP Score	≤0.233	328 (79.6%)	20 (4.9%)	348 (84.5%)
	>0.233	28 (6.8%)	36 (8.7%)	64 (15.5%)
	**Total**	356 (86.4%)	56 (13.6%)	412 (100%)

**Table 4:** Reference points for the Decision Curve Analysis (DCA). The total sample size is N = 412, with a baseline metastatic breast cancer prevalence of 13.6%. All rates and net benefits are expressed as proportions.

Threshold Probability	Positive Rate	True Positive Rate	False Positive Rate	Net Benefit
0.00 (0%)	0.136	0.136	0.864	0.136
0.01 (1%)	0.136	0.136	0.864	0.127
0.02 (2%)	0.136	0.136	0.864	0.118
0.03 (3%)	0.136	0.136	0.864	0.109
0.04 (4%)	0.136	0.136	0.864	0.100
0.05 (5%)	0.136	0.136	0.864	0.090
0.06 (6%)	0.136	0.136	0.864	0.081
0.07 (7%)	0.136	0.136	0.864	0.071
0.08 (8%)	0.136	0.136	0.864	0.061
0.09 (9%)	0.136	0.136	0.864	0.051

## Data Availability

The data that supports the findings of this study is available from the corresponding author upon request.

## Supplementary Materials

The following supporting information can be downloaded at: https://www.mdpi.com/article/10.3390/biomedicines14091960/s1, data: Power analysis result.

## Abbreviations

AUC	Area under the curve
ROC	Receiver operating characteristic
OS	Overall survival
SD	standard deviation
HER2	Human epidermal growth factor receptor 2
ER	Estrogen receptor
PR	Progesterone receptor
MLR	Monocyte-to-lymphocyte ratio
PLR	Platelet-to-lymphocyte ratio
NLR	Neutrophil-to-lymphocyte ratio
PIV	Pan-immune-inflammation value
PNI	Prognostic nutritional index
SII	Systemic immune-inflammation index
HALP	Hemoglobin-Albumin-Lymphocyte-Platelet
IHC	Immunohistochemistry
PET-CT	Positron emission tomography-computed tomography
