# Supplementary material for: Predictive Value of Histopathological Variables and Peripheral Immune-Inflammatory Markers in De Novo Metastatic Breast Cancer: Development and Clinical Utility of the Novel KiTNASP Score
Source: Biomedicines. 2026 Aug 31;14(9):1960. doi: 10.3390/biomedicines14091960 (PMC13604799; doi:10.3390/biomedicines14091960)
Supplement: Supplementary file 1 [file biomedicines-14-01960-s001.zip › biomedicines-4357926-supplementary.pdf]

[1] -- Thursday, August 20, 2026 -- 20:51:05

t tests – Means: Wilcoxon-Mann-Whitney test (two groups)

**Options:**	A.R.E. method		
**Analysis:**	Post hoc: Compute achieved power		
**Input:**	Tail(s)	=	Two
	Parent distribution	=	Normal
	Effect size d	=	1.6655791
	α err prob	=	0.05
	Sample size group 1	=	356
	Sample size group 2	=	56
**Output:**	Noncentrality parameter δ	=	11.3219533
	Critical t	=	1.9660430
	Df	=	391.431
	Power (1–β err prob)	=	1.0000000
